# Tumor mutation burden and circulating tumor DNA in combined CTLA-4 and PD-1 antibody therapy in metastatic melanoma – results of a prospective biomarker study

**DOI:** 10.1186/s40425-019-0659-0

**Published:** 2019-07-12

**Authors:** Andrea Forschner, Florian Battke, Dirk Hadaschik, Martin Schulze, Stephanie Weißgraeber, Chung-Ting Han, Maria Kopp, Maximilian Frick, Bernhard Klumpp, Nicola Tietze, Teresa Amaral, Peter Martus, Tobias Sinnberg, Thomas Eigentler, Ulrike Keim, Claus Garbe, Dennis Döcker, Saskia Biskup

**Affiliations:** 1 Center for Dermatooncology, Department of Dermatology, University Hospital Tuebingen, Liebermeisterstr. 25, 72076 Tuebingen, Germany; 2 Center for Genomics and Transcriptomics (CeGaT) GmbH, Tuebingen, Germany; 3 Practice for Human Genetics, Tuebingen, Germany; 4 Institute for Radiology, Rems-Murr-Kliniken, Winnenden, Germany; 5 Institute for Radiology, University Hospital Tuebingen, Tuebingen, Germany; 6 Portuguese Air Force Health Care Direction, Lisbon, Portugal; 7 Institute for Clinical Epidemiology and applied Biostatistics (IKEaB), Tuebingen, Germany

## Abstract

**Background:**

Metastasized or unresectable melanoma has been the first malignant tumor to be successfully treated with checkpoint inhibitors. Nevertheless, about 40–50% of the patients do not respond to these treatments and severe side effects are observed in up to 60%. Therefore, there is a high need to identify reliable biomarkers predicting response.

Tumor Mutation Burden (TMB) is a debated predictor for response to checkpoint inhibitors and early measurement of ctDNA can help to detect treatment failure to immunotherapy in selected melanoma patients. However, it has not yet been clarified how TMB and ctDNA can be used to estimate response to combined CTLA-4 and PD-1 antibody therapy in metastatic melanoma.

**Patients and methods:**

In this prospective biomarker study, we included 35 melanoma patients with ipilimumab (anti-CTLA-4) and nivolumab (anti-PD-1) therapy. In all patients, a tumor panel of 710 tumor-associated genes was applied (tumor vs. reference tissue comparison), followed by repetitive liquid biopsies. Cell-free DNA was extracted and at least one driver mutation was monitored. Treatment response was evaluated after about three months of therapy.

**Results:**

TMB was significantly higher in responders than in nonresponders and TMB > 23.1 Mut/Mb (TMB-high) was associated with a survival benefit compared to TMB ≤ 23.1 Mut/Mb (TMB-low or TMB-intermediate). Furthermore, a > 50% decrease of cell-free DNA concentration or undetectable circulating tumor DNA (ctDNA), measured by tumor-specific variant copies/ml of plasma at first follow-up three weeks after treatment initiation were significantly associated with response to combined immunotherapy and improved overall survival, respectively. It is noticeable that no patient with TMB ≤ 23.1 Mut/Mb and detectable or increasing ctDNA at first follow-up responded to immunotherapy.

**Conclusion:**

High TMB, > 50% decrease of cell-free DNA concentration, and undetectable ctDNA at first follow-up seem to be associated with response and overall survival under combined immunotherapy. The evaluation of ctDNA and cell-free DNA three weeks after treatment initiation may be suitable for early assessment of efficacy of immunotherapy.

**Electronic supplementary material:**

The online version of this article (10.1186/s40425-019-0659-0) contains supplementary material, which is available to authorized users.

## Introduction

Checkpoint inhibitors such as pembrolizumab, nivolumab, or combination of ipilimumab and nivolumab have significantly improved prognosis of patients with metastatic melanoma. Nevertheless, about 40–50% of the patients do not respond to these treatments and severe side effects such as immune-mediated colitis, hepatitis, pneumonitis, or endocrinological diseases are observed in up to 60% [1–4]. Therefore, there is a high need to identify reliable biomarkers predicting response. Programmed cell death ligand 1 (PD-L1) expression on the tumor cell surface was shown not to be a reliable predictive biomarker for response or survival as checkpoint inhibitors also are effective in patients with PD-L1 negative tumors [5–7]. Furthermore, PD-L1 expression was shown to be inconsistent between primary tumors and metastases and even between metastases within one patient in about 50% of the cases [8]. Among patients treated with pembrolizumab, presence of liver metastases at treatment initiation was found to be associated with significant reduced response rates and progression-free survival, possibly due to reduced CD8^+^ T-cell infiltration at invasive margins [9, 10]. Cutaneous melanoma is a tumor that exhibits a rather high tumor mutation burden (TMB) [11], although there is a very high variation and by far not all melanomas are TMB-high. The correlation between high TMB and response to immunotherapy is not completely clarified but it is potentially a predictor for response [12–18]. It is unclear whether there is an individual TMB cut-off for each type of tumor [19]. So far, there are no precise data on the influence of TMB in melanoma patients with combined immunotherapy on therapy response and survival.

In recent months, there were new insights in the use of circulating tumor DNA (ctDNA) as a predictive marker for early response and prognosis for melanoma patients with checkpoint inhibitors. Patients with persistently elevated ctDNA levels early on treatment had a poor prognosis [20, 21]. Furthermore, increase of ctDNA was found being highly predictive of progressive disease in melanoma patients with BRAF or NRAS mutations [22].

In this prospective study we performed a comprehensive panel sequencing approach using tumor versus normal tissue and repeated liquid biopsies of patients that newly started a combined ipilimumab and nivolumab therapy for metastasized melanoma. The tumor panel comprised 710 tumor-associated genes covering > 2 Mb to reliably calculate TMB [23] to detect all known driver and resistance mutations including copy number variation. Additionally, at least one individual driver mutation was monitored with very high sensitivity in each patient using digital droplet PCR from ctDNA. We aimed to obtain predictive markers for therapy response and survival.

## Materials and methods

### Patients and clinical data

We included metastasized melanoma patients of the Center for Dermatooncology of the University of Tuebingen, who started systemic treatment from January 2018 on and whose tumor tissue was available for tumor sequencing. Written consent for the participation in the study was obtained from all patients and informed consent was also given according to the Gene Diagnostic Law in Germany. Response was calculated as percentage of responses among all patients and was assessed by comparison of patients’ CTs before initiation of combined immunotherapy and first staging thereafter. The baseline tumor load and response to therapy was assessed by oncologic experienced radiologists according to RECIST 1.1. [24]. To obtain a score for tumor load, the five largest lesions in each CT were measured and summed to a “CT score”. In one patient with stable disease in the sum at first staging, the second staging three months later was also considered, resulting in progressive disease (PD). Two patients who had no follow-up imaging due to rapid clinical progression as assessed by the physician were classified as having progressive disease. Two patients died due to other reasons than progressive disease, one suffered myocardial infarction, the other one had pre-existing cardiac disease and died of decompensated heart failure. Both patients had exhibited an excellent response to treatment. They were considered as censored cases for the melanoma specific survival analyses.

The ethics committee of the Ärztekammer Baden-Württemberg and the local ethics committee of the Eberhard Karls University approved this study (approval numbers F-2016-010 and 827/2018BO2).

The formalin-fixed paraffin-embedded tissue that had been used for sequencing was the latest available tissue, usually metastases that had been removed recently. In 21 patients, PD-L1 status had been determined as part of the clinical routine. All samples were stained with an antibody against PD-L1 (28–8, 1100, Abcam, Cambridge, UK). Primary antibody detection was performed using the OptiView DAB IHC detection kit (Ventana). Blood samples for ctDNA were taken at the same time as the laboratory controls, which were indicated in context of the immunotherapy.

In this evaluation, we included 35 patients with combined ipilimumab and nivolumab treatment and assessment of treatment response until August 17th 2018.

### Cell-free DNA and tumor sequencing

In all patients, a tumor panel of 710 tumor-associated genes was analyzed (tumor vs. reference tissue comparison), followed by repetitive liquid biopsies every 3–4 weeks.

#### Tumor panel analysis

From EDTA blood and tumor samples (primary tumor, metastasis), genomic DNA was isolated according to the manufacturers’ instructions using QIAamp DNA Blood Maxi Kit on a QiaSymphony instrument (Qiagen, Hilden, Germany) or blackPREP FFPE DNA Kit (Analytik Jena, Jena, Germany), respectively. DNA isolation of tumor DNA was performed after macro dissection by pathologist or neuropathologist. DNA quantity and quality were determined using Qubit® Fluorometer (Thermo Fisher Scientific, Dreieich, Germany) and Fragment Analyzer (AATI, Heidelberg, Germany), respectively. All coding regions and flanking intronic regions of 710 genes were enriched using Agilent in-solution bait-hybridization technology. For sequencing we used either Illumina HiSeq2500, HiSeq4000, or NovaSeq6000 systems (Illumina, San Diego, USA).

#### Isolation of plasma and cell-free DNA and ddPCR analysis

Whole blood was collected in either EDTA tubes or in cell-free BCT tubes (Streck, cat. no. 218992). From these, plasma was isolated by a double centrifugation protocol (1900 g, 10 min, 4 °C and 16,000 g, 10 min, 4 °C). Cell-free DNA was extracted from 4 ml plasma using MagMAX Cell-Free DNA Isolation Kit (ThermoFisher, cat. no. A29319). Quality and quantity of cell-free DNA were analyzed using High Sensitivity NGS Fragment Analysis Kit (AATI, cat. no. DNF-474) and Qubit dsDNA HS Assay Kit (ThermoFisher, cat. no. Q32854), respectively.

For digital droplet PCR, dual-probe TaqMan assays were designed to determine the presence of somatic mutations in cell-free DNA which were known from previous tumor tissue sequencing. If possible, assays were designed for therapeutically relevant mutations identified in the autologous tumor tissue. For assay design, the target region covering 80 base pairs (bp) upstream and downstream of a variant were retrieved from University of California, Santa Cruz (UCSC) Genome Browser (human genome: GRCh 38/hg38) [25]. A search by the Basic Local Alignment Search Tool (BLAST) was conducted to identify potentially present pseudogenes or other regions displaying strong homology to the target region [26]. Within the target region, common single nucleotide variants (AF ≥ 1%), homologous regions as well any patient specific germline and somatic variants were masked and the variant position marked. The marked target sequence was pasted into the online design tools of Thermo Scientific or BioRad for automated assay design. In some cases, commercially available predesigned assays were ordered from Thermo Scientific or BioRad. A list of all assays used can be found in Additional file 1: Table S1.

All ddPCR-based cell-free DNA analyses were performed (BioRad QX200 Droplet Generator and Reader, BioRad, Munich, Germany) and reported according to the digital MIQE guidelines [27] [28]. Numbers of mutant and wild-type DNA copies were calculated, and mutant allele frequencies were calculated by dividing the number of mutant DNA copies by the total number of DNA copies in the plasma sample. DNA from patients’ tumor tissue and reference DNA (obtained from Coriell Institute) were used positive and negative controls, respectively.

#### Bioinformatics

For NGS analysis, sequencing reads were demultiplexed using Illumina bcl2fastq (1.8.2). Adapter sequences were removed with Skewer 0.1.116 and the trimmed reads mapped to the human reference genome (hg19) using the Burrows Wheeler Aligner (BWA-mem 0.7.2). Reads mapping to more than one location with identical mapping scores were discarded. Duplicates resulting from PCR amplification and nonuniquely mapping reads were removed (CeGaT proprietary software). Variants were called and technical artefacts removed (CeGaT proprietary software). The resulting variants were annotated based on several internal and external databases.

For each patient, both, tumor tissue as well as reference tissue were analyzed and the data compared to reliably distinguish somatic mutations from germline variants.

TMB was defined as the number of somatic single nucleotide variants, InDel-, and essential splicing changes in the complete coding region (exome) and reported as mutations (Mut) per one million coding bases (Mb). To compute tumor mutation burden, first the somatic variants affecting the protein-coding regions of all sequenced genes (both synonymous as well as non-synonymous) with a minimum variant frequency of 10% were counted. Variants identified by 710 gene panel sequencing were split into driver and passenger mutations and the resulting two counts used to estimate the number of somatic variants in the whole exome. For this estimation, passenger mutations were assumed to occur with equal density in all known genes, i.e., their number was scaled up relative to the difference between gene panel size and whole exome size. Driver mutations were assumed to be limited to tumor-associated genes, and their number was not scaled up. The estimated total count of both passenger and driver mutations was normalized to the size of the complete coding exome. The classification of the determined mutation load per encoding Megabase DNA was carried out in the categories “low” (< 3.3 Mut/Mb) “intermediate” (3.3–23.1 Mut/Mb) and “high” (> 23.1 Mut/Mb) [29] [14].

### Statistical analysis

Statistical analysis was performed using the statistical program for social sciences SPSS Version 25 (IBM, New York, United States) and R (Version 3.4.4, R Core Team, 2018). Descriptive statistics were used to describe the study collective. The distribution of TMB in responders and non-responders was compared using the nonparametric Wilcoxon Rank-Sum test as implemented in R. Differences between groups were tested using the Exact Fisher test and the Exact Version of the Chi-Square trend test for categorical data (response and comparisons between potential predictors) and the Log rank test (melanoma specific survival). Survival curves were generated according to the Kaplan-Meier method: Survival time was defined as the time between first cycle of ipilimumab+nivolumab and melanoma specific death, or censored at the last date of patient contact. It was not possible to perform multivariate (logistic or Cox) regression analysis, probably due to the relatively small number of cases. None of the models with two predictors converged using the iterative algorithm of SPSS for maximum likelihood estimation. Thus, for the relevant pairs of predictors (TMB in combination with detection of ctDNA, increase of ctDNA or cell-free DNA at first follow-up, liver metastasis, or sex), we built combined variables with three to six categories each. Then we performed the Exact Version of the Chi-Square trend test for categorical data and Kaplan Meier analysis with Log rank test for these combined variables. The level of significance was 0.05 (two-sided) in all analyses. Adjustment for multiple testing was not performed.

## Results

### Patient cohort

We prospectively included advanced melanoma patients that started a new systemic treatment from January 2018 on and whose tumor tissue was available for tumor sequencing. In this evaluation, we only included 35 patients that started treatment with ipilimumab and nivolumab in the time from January 8th to May 24th 2018. The following melanoma subtypes were involved: 20 (57%) cutaneous, 6 (17%) occult, 4 (11%) uveal, 3 (9%) acral and 2 (6%) mucosal melanomas. About half of the patients were female (46%) and the largest part of the patients (63%) started ipilimumab and nivolumab as their first line systemic treatment. 10 patients (29%) had been treated with targeted therapy before and 3 (9%) with PD-1 antibodies. 89% of the tissues sequenced were therapy naïve, 43% issued from lymph node metastases, 51% from other than lymph node metastases and in 6% the primary melanoma was used for sequencing as no metastasis was accessible. Median time until the first staging was 69 days (IQR 49–80), median follow-up since start of combined immunotherapy was 213 days (IQR 175–272).

PD-L1 status was not determined in this prospective study, but in the routine of clinical care it was assessed in 21/35 patients (60%). PD-L1 < 1% was found in 11 patients (31%), PD-L1 ≥ 1% in 10 patients (29%). In 14 patients (40%) there was no determination of PD-L1 expression.

17 patients (49%) completed 4 cycles of combined immunotherapy, 8 (23%) received 3 cycles, 7 (20%) 2 cycles and 3 patients only received one cycle. One of these three patients had rapid progression and died. Another suffered severe myocarditis and diabetes mellitus and therefore continued nivolumab alone. A third patient refused further treatment.

Baseline lactate dehydrogenase (LDH) was elevated in 40% of the patients and about one third of the patients had liver or brain metastases respectively. 63% of the patients suffered adverse events (AE) of CTCAE grade 3 or 4. Median time until onset of these AE was 42 days (IQR 21–61) (Table 1). Among all non-responders, 12 suffered adverse events grade 3 or 4.

**Table 1:** Clinical characteristics of the cohort

Patients` characteristics (*n* = 35)	median	IQR	range
Age at first diagnosis of melanoma (years)	55	47–70	17–79
Time between primary diagnosis and first distant metastasis (months)	17	5–43	0–241
Time between start of combined immunotherapy and first staging (days)	69	49–80	12–108
Time between start of combined immunotherapy and onset of severe adverse events (days)	42	21–61	11–126
		no. patients	%
Sex			
Female		16	46
Male		19	54
Melanoma type			
Cutaneous		20	57
Occult		6	17
Uveal		4	11
Acral		3	9
Mucosal		2	6
BRAF mutation			
BRAF v600 positive		16	46
BRAF v600 negative		19	54
Systemic treatment before combined immunotherapy			
None – combined immunotherapy as first line		22	63
Targeted therapy		10	29
PD1 antibody		3	9
LDH at start of combined immunotherapy			
LDH elevated		14	40
LDH normal		21	60
Metastasis at start of combined immunotherapy			
Presence of lung metastases		19	54
Presence of brain metastases		12	34
Presence of liver metastases		10	29
AJCC (2017) stage			
M1a/M1b		11	31
M1c		12	34
M1d		12	34
Cycles of combined immunotherapy			
1 cycle		3	9
2 cycles		7	20
3 cycles		8	23
4 cycles		17	49
Response to combined immunotherapy			
Response		15	43
*-- complete response*		*4*	*11*
Progressive disease		20	57
Adverse events CTCAE 3–4		22	63
Origin of tissue sequenced			
Lymph node metastasis		15	43
Other metastasis		18	51
Primary melanoma		2	6
Status of tissue sequenced			
Tissue therapy naïve		31	89
Tissue pretreated		4	11

### Cell-free DNA and tumor sequencing

Results of tumor sequencing were obtained for all 35 patients. Due to qualitative deficiencies, no TMB could be determined in 5 samples: in two samples the material did not yield a sequencing library of sufficient complexity resulting in very low coverage. One sample was contaminated. In two samples the tumor content was too low.

Median tumor mutation burden was 4.7 Mut/Mb (IQR 2–17). Categorized in 3 TMB groups as published before [14, 29], there were 11 (37%) classified low (< 3.3 Mut/Mb), 13 (43%) intermediate (3.3–23.1 Mut/Mb) and 6 (20%) high (> 23.1 Mut/Mb).

Cell-free DNA was collected at baseline for 34 patients. At time point 2, ddPCR could be obtained from 32 patients, at time point 3 from 28 patients, and at time point 4 from 25 patients. Median time between baseline and first follow-up cell-free DNA samples was 23 days (IQR 21–29). Somatic mutations in the following genes were analyzed in the patients’ plasma: *BRAF, CDK4, GNAQ, JAK2, KRAS, MAP2K1, NF1, NRAS, STAT1* (mutations indicated in Additional file 1: Table S1).

### Published cutoffs for high TMB are supported by our data

We sought to determine whether our cohort supports previously published thresholds stratifying patients into TMB-high and not-high groups. A recently published study including 321 melanoma patients suggested a threshold of 30.7 Mut/Mb using the MSK-Impact panel [19]. While an earlier publication set the threshold independent of the cancer type at 23.1 Mut/Mb using the FoundationOne panel [14], based on several hundred samples, of which 121 melanoma samples.

In our cohort, the mean TMB was 43.2 (median 23.1) for responders and 4.81 (median 3.4) for non-responders, respectively. The highest TMB of a non-responder was 17.3. As a result, all thresholds between 18 and 31 Mut/Mb provided equally significant stratification (data not shown). Samstein and colleagues set their threshold of 30.7 to classify 20% of cases as “TMB-high” [19]. Using this threshold in our cohort, 6/30 (20%) of patients are classified as “high”. Our cohort thus supports both published thresholds, although TMB was determined using different methods. However, it should be noted that larger cohorts allow a more accurate threshold determination.

### TMB, cell-free DNA and ctDNA are associated with response

**Fig. 1:**
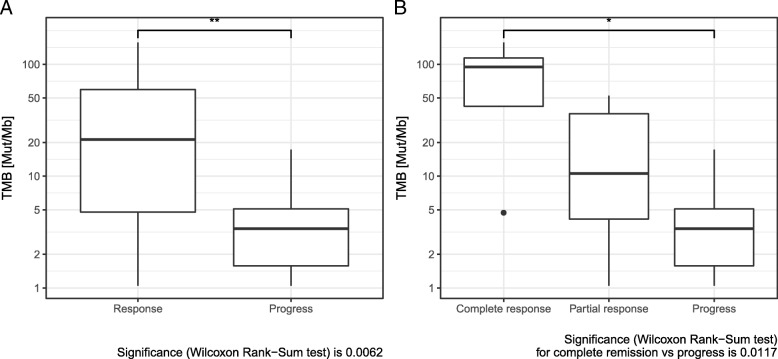
**a** Comparison of tumor mutation burden (TMB) in responders and non-responders to combined immunotherapy. **b** Comparison of tumor mutation burden (TMB) in complete responders, partial responders, and non-responders to combined immunotherapy

Comparing median TMB of responders and non-responders revealed a significant difference. Median TMB was significantly higher in patients with response to immunotherapy (Fig. 1a). When complete responders were evaluated separately, an even higher TMB value in this subgroup became evident (Fig. 1b). Response to immunotherapy also correlated significantly to TMB, classified in the three categories: TMB high (> 23.1 Mut/Mb), intermediate (3.3–23.1 Mut/Mb) and low < 3.3 Mut/Mb) [14, 29]. In addition, there was a significant difference in response, when classifying TMB into 2 classes (high versus intermediate+low) (Table 2). There was a significant sex difference between the groups TMB > 23.1 Mut/Mb and TMB ≤ 23.1 Mut/Mb (Additional file 2: Table S2a): all patients with TMB > 23.1 Mut/Mb were male.

**Table 2:** Impact of patients’ and disease characteristics on treatment response and overall survival since the beginning of combined immunotherapy

	Total *n* = 35	Responder *n* = 15	Non-Responder *n* = 20	Response *P* value	Overall survival *P* value
TMB [Mut/Mb]					
> 23	**6**	**6**	**0**	**0.002** ^1*^	0.06^2^
≤ 23	**24**	**6**	**18**		
ctDNA [copies/ml] detection^5^					
Not detectable	**14**	**10**	**4**	**0.011** ^1*^	**0.006** ^2*^
Detectable	**18**	**4**	**14**		
ctDNA [copies/ml] increase^5^					
Not increasing	**20**	**12**	**8**	**0.008** ^1*^	**0.03** ^2*^
Increasing	**11**	**1**	**10**		
Cell-free DNA [ng/ml]^6^					
Decrease > 50%	**7**	**5**	**2**	**0.022** ^3*^	**0.005** ^4*^
Stable	**15**	**7**	**8**		
Increase > 50%	**9**	**1**	**8**		
LDH baseline elevated					
No	**21**	**11**	**10**	0.296^**1**^	**0.001** ^2*^
Yes	**14**	**4**	**10**		
Targeted therapy before					
No	**25**	**12**	**13**	0.458^**1**^	**0.001** ^2*^
Yes	**10**	**3**	**7**		
Sex					
Male	**19**	**11**	**8**	0.087^**1**^	**0.005** ^2*^
Female	**16**	**4**	**12**		
Liver metastasis baseline					
No	**25**	**14**	**11**	**0.022** ^1*^	**0.013** ^2*^
Yes	**10**	**1**	**9**		
PD-L1 Expression					
≥ 1%	**10**	**6**	**4**	0.080^1^	0.772^2^
< 1%	**11**	**2**	**9**		

* significant (in bold).

^1^Exact Test of Fisher

^2^Log rank test

^3^Exact Chi-Square Test for Trend (Monte Carlo Simulation)

^4^Log rank test for Trend

^5^ctDNA measured by tumor-specific variant [copies/ml plasma] at first follow-up after start of combined immunotherapy

^6^Cell-free DNA [ng/ml plasma] at first follow-up after start of combined immunotherapy

While cell-free DNA can be found in blood plasma at baseline concentrations in healthy individuals and fluctuates with such factors as, e.g., physical exercise, concentrations have been reported to be elevated in patients with progressive disease as well as during the initial stages of a successful tumor therapy, when a large number of tumor cells perish [30]. We measured cell-free DNA concentrations (i.e., the concentration of cell-free DNA regardless of whether such DNA was tumor-derived or not) as well as tumor-specific variant copies/ml of plasma for each patient quantified by digital droplet PCR (ddPCR). The tumor-derived cell-free DNA, i.e. circulating tumor DNA (ctDNA) is measured as tumor specific variant copies/ml of plasma. ddPCR is a very sensitive method and allows to detect ctDNA at allele frequencies of > 0.2% from 5 ng of DNA if three independent observations (droplets) are set as threshold of detection.

**Fig. 2:**
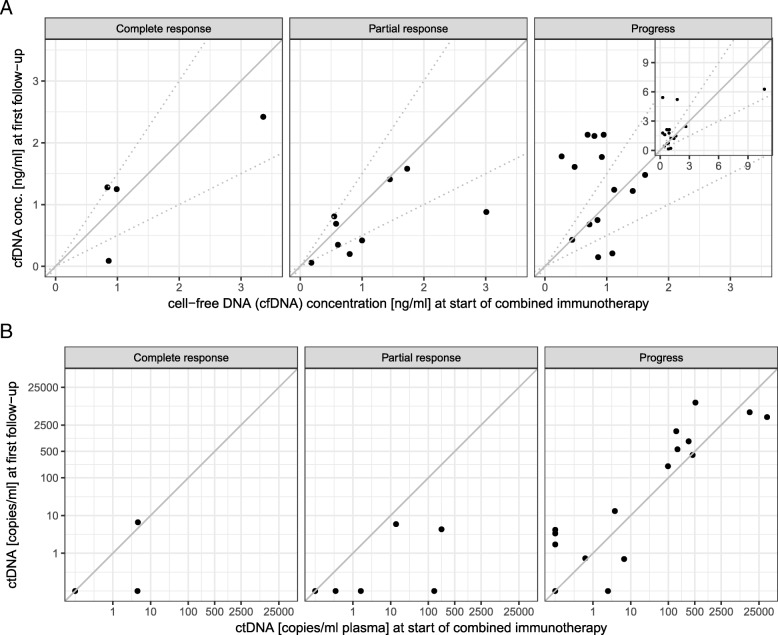
**a** Cell-free DNA concentrations at start of combined immunotherapy (x axis) and at first follow-up (3–4 weeks later, y axis). Patients were classified into three groups, depending on the change in their cell-free DNA concentration as increasing (increase of more than 50%), decreasing (decrease of more than 50%), or stable. The respective thresholds are marked by broken lines. Increase of cell-free DNA is observed more frequently in non-responders. The four highest values can be seen in the inserted image in the upper right corner. **b** ctDNA, measured by tumor-specific variant copies/ml of plasma at start of combined immunotherapy (x axis), and at first follow-up (3–4 weeks later, y axis). Increase of ctDNA is almost only observed in progressive patients. Please note that multiple patients had undetectable ctDNA at both time points and are not visible in the plot as separate points due to overplotting (2 for complete response, 4 for partial response, 3 for progress)

Increasing cell-free DNA concentration was observed more often in progressive patients (Fig. 2a, Table 2). Accordingly, a decrease > 50% of cell-free DNA concentration at first follow-up, 3 weeks after treatment initiation, was significantly associated with response to combined immunotherapy (Table 2).

Increase in ctDNA copies at first follow-up occurred almost exclusively in progressive patients. In all but one therapy responders (with TMB high), ctDNA decreased or remained stable already during the first follow-up control (Fig. 2b, Table 2). Likewise, ctDNA remaining or becoming undetectable at first follow-up was significantly more common in responders. Only four of 18 patients with detectable ctDNA at first follow-up responded to combined immunotherapy (Table 2). In 8 of the 12 progressive patients suffering adverse events grade 3 or 4, ctDNA was detectable at first follow-up*.*

It is remarkable that ctDNA was detectable even with very low tumor load in baseline or follow-up CT. Also, a number of patients with low initial CT scores but progress at first follow-up already showed high ctDNA counts at the start of therapy (Additional file 3: Figure S3).

Regarding the combined variables (Table 3), no patient with TMB ≤ 23.1 Mut/Mb and either ctDNA increase or ctDNA detectable or cell-free DNA increase of > 50% at first follow-up responded to immunotherapy. If patients with TMB ≤ 23.1 Mut/Mb were responders, they had neither ctDNA increase nor ctDNA detectable at first follow-up.

**Table 3:** Impact of patients’ and disease characteristics (combined variables) on treatment response and overall survival since the beginning of combined immunotherapy

	Total *n* = 35	Responder *n* = 15	Non-Responder *n* = 20	Response *P* value	Overall survival *P* value
Combination of TMB [Mut/Mb] and ctDNA [copies/ml] detection^3^					
TMB > 23 Mut/Mb + ctDNA not detectable	**3**	**3**	**0**	**< 0.0001** ^1*^	**0.005** ^2*^
TMB > 23 Mut/Mb + ctDNA detectable	**3**	**3**	**0**		
TMB ≤ 23 Mut/Mb + ctDNA not detectable	**8**	**5**	**3**		
TMB ≤ 23 Mut/Mb + ctDNA detectable	**13**	**0**	**13**		
Combination of TMB [Mut/Mb] and ctDNA [copies/ml] increase^3^					
TMB > 23 Mut/Mb + ctDNA not increasing	**4**	**4**	**0**	**< 0.0001** ^1*^	**0.032** ^2*^
TMB > 23 Mut/Mb + ctDNA increasing	**1**	**1**	**0**		
TMB ≤ 23 Mut/Mb + ctDNA not increasing	**12**	**5**	**7**		
TMB ≤ 23 Mut/Mb + ctDNA increasing	**9**	**0**	**9**		
Combination of TMB [Mut/Mb] and cell-free DNA [ng/ml]^4^					
TMB > 23 Mut/Mb + cfDNA decrease > 50%	**3**	**3**	**0**	**0.001** ^1*^	**0.016** ^2*^
TMB > 23 Mut/Mb + cfDNA stable	**1**	**1**	**0**		
TMB > 23 Mut/Mb + cfDNA increase > 50%	**1**	**1**	**0**		
TMB ≤ 23 Mut/Mb + cfDNA decrease > 50%	**3**	**1**	**2**		
TMB ≤ 23 Mut/Mb + cfDNA stable	**11**	**4**	**7**		
TMB ≤ 23 Mut/Mb + cfDNA increase > 50%	**7**	**0**	**7**		
Combination of TMB [Mut/Mb] and liver metastases					
TMB > 23 Mut/Mb + no liver metastases	**6**	**6**	**0**	**< 0.0001** ^1*^	**0.018** ^2*^
TMB ≤ 23 Mut/Mb + no liver metastases	**14**	**5**	**9**		
TMB ≤ 23 Mut/Mb + liver metastases	**10**	**1**	**9**		
Combination of TMB [Mut/Mb] and sex					
TMB > 23 Mut/Mb + male	**6**	**6**	**0**	**0.002** ^1*^	**0.010** ^2*^
TMB ≤ 23 Mut/Mb + female	**9**	**2**	**7**		
TMB ≤ 23 Mut/Mb + female	**15**	**4**	**11**		

* significant (in bold).

^1^Exact Chi-Square Test for Trend (Monte Carlo Simulation)

^2^Log rank test for Trend

^3^ctDNA measured by tumor-specific variant [copies/ml plasma] at first follow-up after start of combined immunotherapy

^4^Cell-free DNA [ng/ml plasma] at first follow-up after start of combined immunotherapy

### TMB, cell-free DNA and ctDNA are associated with overall survival

**Fig. 3:**
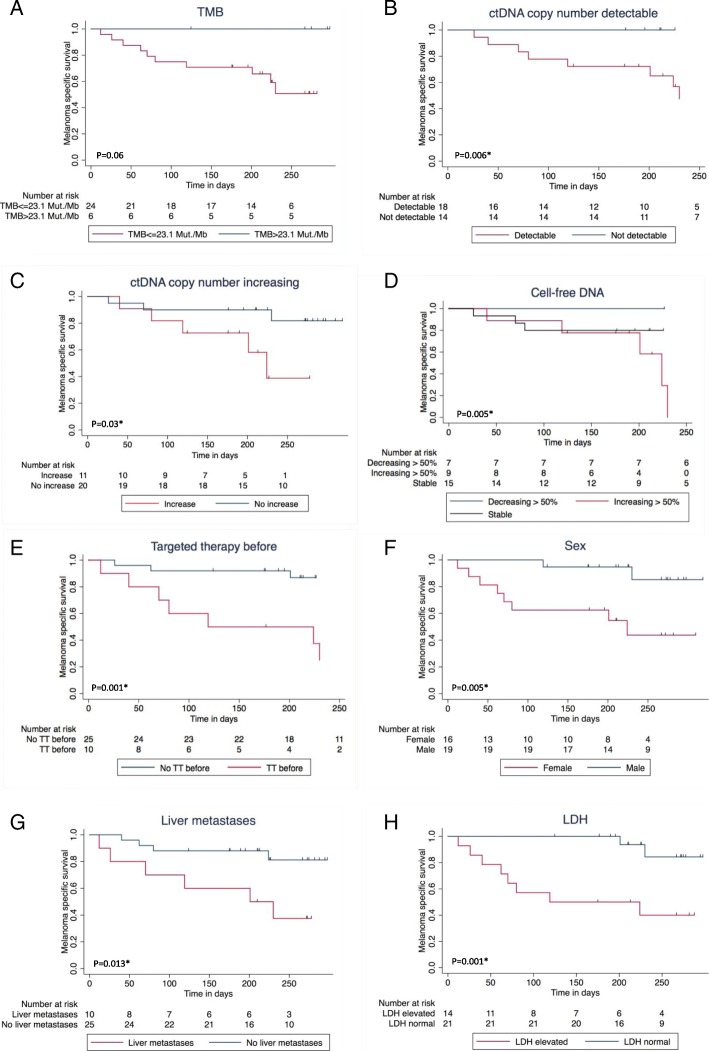
Impact of baseline patients’ and disease characteristics on overall survival since the beginning of combined immunotherapy. ^1^Log rank test / ^2^Log rank test for Trend. *significant. **a** Tumor mutation burden (TMB) > 23.1 Mut/Mb vs. TMB ≤ 23.1 Mut/Mb, *p* = 0.06^1^. **b** ctDNA measured by tumor-specific variant copies/ml of plasma detectable vs. undetectable at first follow-up, *p* = 0.006*^1^. **c** ctDNA measured by tumor-specific variant copies/ml of plasma increasing vs. not increasing at first follow-up, *p* = 0.03*^1^. **d** Cell-free DNA decrease > 50% vs. stable vs. increase > 50%, *p* = 0.005*^2^. **e** Targeted treatment (TT) before start of combined immunotherapy vs. no TT before, *p* = 0.001*^1^. **f** Men vs. women, p = 0.005*^1^. **g** Liver metastasis baseline vs. no liver metastasis baseline, *p*=0.013*^1^. **h** LDH baseline normal vs. elevated, *p* = 0.001*^1^

In the mono-variate overall survival analysis according to Kaplan-Meier, > 50% increasing cell-free DNA, detectable or increasing ctDNA at first follow-up were significant negative influence factors on overall survival. Furthermore, patients with high TMB showed a trend towards prolonged survival (Fig. 3a-d) (Table 2).

**Fig. 4:**
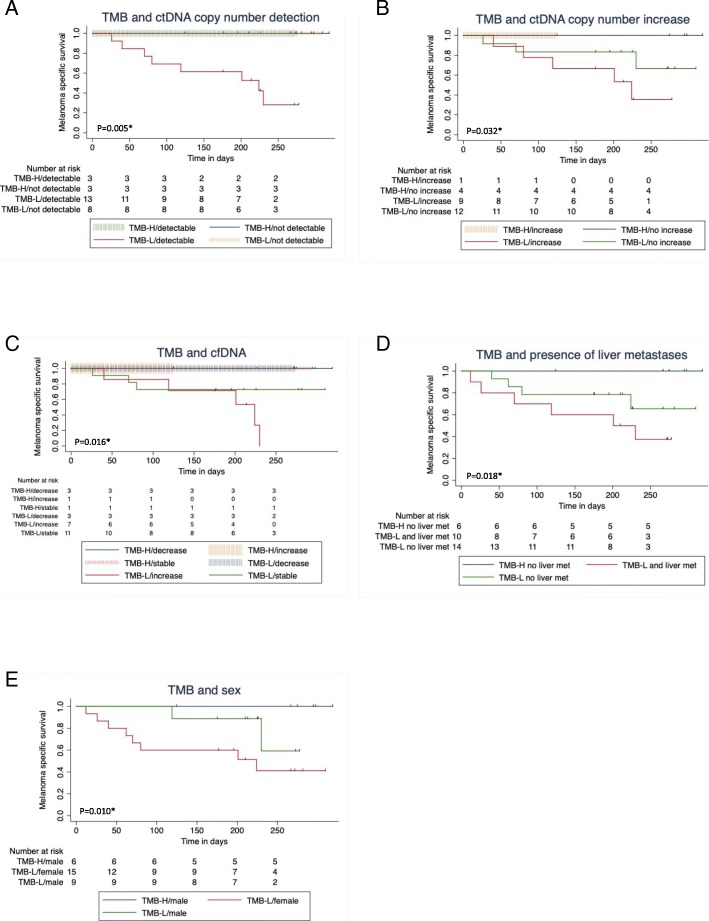
Impact of combined variables of TMB on overall survival since the beginning of combined immunotherapy. TMB > 23.1 Mut/Mb (TMB-H) TMB ≤ 23.1 Mut/Mb (TMB-L). Log rank test for Trend. *significant. **a** Tumor mutation burden (TMB) and ctDNA measured by tumor-specific variant copies/ml of plasma detectable vs. undetectable at first follow-up p = 0.005*. **b** Tumor mutation burden (TMB) and ctDNA measured by tumor-specific variant copies/ml of plasma increasing vs. not increasing, *p* = 0.032*. **c** Tumor mutation burden (TMB) and cell-free DNA decrease > 50% vs. stable vs. increase > 50% at first follow-up, *p* = 0.016*. **d** Tumor mutation burden (TMB) and presence of liver metastases, *p* = 0.018*. **e** Tumor mutation burden (TMB) and sex, *p* = 0.010*

Regarding the combined variables (Table 3), overall survival was worse in patients with TMB ≤ 23.1 Mut/Mb and either ctDNA increase or ctDNA detectable or cell-free DNA increase of > 50% at first follow-up. If TMB was low, survival was improved if there was no increase or detection of ctDNA at first follow-up (Fig. 4a-c).

### Other prognostic factors

The presence of liver metastases also had a significant negative impact on response (Table 2). Furthermore, there was a trend towards lower response rate for patients with elevated LDH at the beginning of combined immunotherapy, for women, for patients that had been under treatment with targeted therapy before starting ipilimumab and nivolumab, and for patients with PD-L1 expression < 1% (Table 2). Only 2/11 (18%) patients with PD-L1 expression < 1% responded, but 6/10 (60%) with at least 1% PD-L1 positivity. Concerning the presence of liver metastasis, LDH elevation, PD-L1 expression, and targeted therapy before combined immunotherapy, there were no significant differences between the two TMB groups (Additional file 2: Table S2a). It is noteworthy that patients with TMB ≤ 23.1 Mut/Mb and liver metastases had an even worse prognosis than those without liver metastases (Fig. 4d). Elevated baseline LDH, presence of liver metastases baseline, pre-treatment with targeted therapy, and female sex were significant negative influence factors on overall survival in our cohort (Fig. 3e-h). The survival difference between men and women (Fig. 3f) cannot completely be explained by the fact that all TMB-high patients were male. Even in TMB-low patients, women had a worse prognosis (Fig. 4e).

## Discussion

In our prospective biomarker study, response and OS of melanoma patients treated with combined immunotherapy could be shown to be positively associated with high TMB, > 50% decreasing cell-free DNA or undetectable ctDNA at first follow-up. Combination of TMB and cell-free or ctDNA were even more important. No patient with TMB ≤ 23.1 Mut/Mb and either ctDNA increase or ctDNA detectable, or cell-free DNA increase of > 50% at first follow-up responded to immunotherapy. Up to now it has only been reported that higher TMB and decreasing ctDNA in melanoma patients is significantly associated with response and overall survival (OS) to checkpoint inhibitor monotherapy, but not in detail to combined immunotherapy and not the combination of TMB and ctDNA [12, 13, 15, 19, 20, 22, 31, 32].

Melanomas belong to the tumors with the highest mutation burden, most probably attributed to DNA damage by UV light [11, 29, 33]. Within all melanomas, cutaneous melanomas harbor a significantly higher mutation load than melanomas at UV-protected sites such as acral melanoma, mucosal melanoma, or uveal melanoma [29, 34–36]. A positive correlation between high mutation load of the tumor and better response to immunotherapy is not surprising: the more mutations a tumor genome contains, the higher the chance that neo-antigens are presented on its surface, which render the tumor more recognizable by T cells [37, 38]. In lung cancer it has recently been shown that higher TMB correlated with better response to combined immunotherapy [39]. In melanoma such significant correlation has not been shown yet.

Kaplan-Meier curves display a trend for better survival for TMB > 23.1 Mut/Mb compared to TMB ≤ 23.1 Mut/Mb. When dealing with TMB, it has to be mentioned that remarkably rather different thresholds have been chosen. Snyder and colleagues found significant improved OS in ipilimumab-treated melanoma patients with TMB > 100, determined by whole exome sequencing [13]. In contrast, Morrison and colleagues failed to reveal significant differences in OS of melanoma patients with immunotherapy by applying a threshold which was determined by doubling the median and amounted to 7.1 Mut/Mb [40]. For completeness it should be mentioned that we have also made the evaluation with a TMB cut-off of 2 times the median (9.4) for comparison purposes. There was also a significant association with the response rate, but no association with OS. Recently it has been reported that among cancer patients with immunotherapy, those with higher TMB (the highest 20% of each cancer type) had a better survival. Regarding melanoma patients, the cut-off was 30.7 Mut/Mb [19]. Interestingly, all of our patients in the group > 23.1 Mut/Mb were also above 30.7 Mut/Mb. It is also noteworthy that this group of patients with TMB > 23.1 accounts for the 20% highest TMB values in our cohort. Therefore our results fit very well with those of Samstein et al. [19].

Rowe and colleagues recently reported a sensitivity and specificity of ctDNA in the detection of melanoma metastases of 87 and 100%. A higher tumor load was positively associated with the detection of ctDNA [21, 41]. The presence of visceral metastases such as liver metastases had been shown to increase the detectability of ctDNA in the plasma, in contrast to metastases exclusively confined to pulmonal or cerebral localization [21].

In our cohort, there was no significant difference in the Exact Test of Fisher between the presence of liver metastases and neither baseline ctDNA (data not shown), nor detectable ctDNA or increasing ctDNA at first follow-up (Additional file 2: Table S2b-c). However, the number of patients with liver metastases in our cohort was low and a strong tendency towards correlation cannot be dismissed. We were able to collect early follow-up cell-free DNA and ctDNA approximately every three weeks and the comparison of the follow-up values to baseline allowed us to assess a tendency for response.

It is remarkable that no patient with TMB ≤ 23.1 Mut/Mb and either ctDNA increase or ctDNA detectable or cell-free DNA increase of > 50% at first follow-up responded to immunotherapy. These results offer the possibility of estimating a therapy response at a very early time point, already at first follow-up. In case of severe adverse events early after treatment initiation, this could be helpful in the decision whether to continue immunotherapy or not. In our cohort, 67% of the progressive patients suffering at least grade 3 adverse events had detectable ctDNA at the first follow-up.

In other studies, the predictive value of response in the first staging was significant for melanoma patients with either no detection of ctDNA baseline or with positive baseline ctDNA becoming undetectable within 12 weeks of immunotherapy. Furthermore, ctDNA was shown to be superior to other baseline parameters, such as ECOG performance status, LDH, or tumor burden [20]. However, in some of these studies liquid biopsies were obtained at different time points, or less than 50% of blood samples were available at time point 3–4 weeks after the start of therapy [20]. Ashida and colleagues reported that decreasing levels of ctDNA already three weeks after immunotherapy initiation were found in all melanoma patients responsive to pembrolizumab but not in the progressive ones [32]. These results corroborate our results, pointing out that one can already evaluate treatment response at a very early stage. In another study an early therapy response to immunotherapy by means of PETCT has been determined, changing the way of thinking that success or failure of an immunotherapy should be evaluated at the earliest 12 weeks after initiation [42]. However we and Ashida found that already the very first follow-up of cell-free DNA / ctDNA within 3–4 weeks after therapy initiation could provide information about response / non-response.

We decided to include analysis of cfDNA (i.e., total cell-free DNA, not limited to tumor-derived DNA only) in our evaluation as the amount of cfDNA per ml plasma can be determined more readily than the number of tumor-derived copies of DNA (ctDNA). This is particularly important when no patient-specific ddPCR assay is available. cfDNA levels can thus be used as an early marker and should also be considered in follow-up analyses even if ctDNA is determined by ddPCR as well. Successful therapy can suppress a tumor clone carrying the queried mutation while other clones without that mutation may thrive. In ctDNA analysis of the targeted mutation, this would result in a reduction of the observed ctDNA with simultaneously increasing concentration cfDNA. Using both markers together can thus help to increase the sensitivity of detecting progress.

We were able to offer the possibility of detecting genes other than BRAF and NRAS in the plasma. We used either commercially available or individually designed ddPCR assays to detect individual somatic variants identified in previous tumor tissue sequencing (Additional file 1: Table S1). This means that for almost any patient, even triple wildtype patients, an individual variant could be monitored by using liquid biopsy. In the future the sensitivity of such analysis may be increased by raising the cell-free DNA input amount per ddPCR analysis (here 5 ng was used for most cases) and by extending this analysis to several known somatic mutations per patient with a potential huge impact in determining treatment success if this becomes available for every patient in a clinical routine.

Our observed sex-specific difference in ORR and OS of melanoma patients treated by immune checkpoint inhibitors are further strengthened by a large meta-analysis including 3632 melanoma patients. This study revealed quite obviously that male metastasized melanoma patients treated by immunotherapy had a better outcome than female melanoma patients. The pooled survival rate for men was double that for women [43]. It has to be considered that Gupta and colleagues reported a significant higher median TMB in male melanoma patients than in female ones. Of note, melanoma was the only tumor in which they found this sex difference, also in lung carcinoma there was no sex difference in TMB [44]. On the other hand, Xiao and colleagues also found higher TMB in male patients with lung adenocarcinoma [45]. Indeed, a higher TMB in male melanoma patients could provide an explanation for the different outcome between male and female melanoma patients treated by immune checkpoint inhibitors. Nevertheless, even if there was a significant sex difference in the two TMB groups of our cohort (Additional file 2: Table S2a), worse prognosis for women was still obvious when comparing only TMB low patients (Fig. 4e). Likewise, Goodman and colleagues found a significant sex difference in their cohort of melanoma and NSCLC patients: 76% of the patients with TMB high were male (*p* = 0.035). In multivariate analysis, TMB remained a significant influence factor on response to immunotherapy, not sex [15].

The negative impact of liver metastases on response and OS of patients with anti-PD-1 therapy has already been shown, but again not concerning combined immunotherapy [9, 46, 47]. To the best of our knowledge, this is the first study to demonstrate both, better ORR and OS in patients without liver metastases. Remarkably, ORR and OS of patients with TMB ≤ 23.1 Mut/Mb were even worse if additional liver metastases were present (Fig. 4d).

Combined immunotherapy after progress under targeted therapy was not successful in our cohort. This emphasizes the high medical need to carefully decide about first line treatment. Nevertheless it cannot be excluded that the patients that had been treated with targeted therapy before had a higher initial tumor load at the onset of metastasis and thus a worse prognosis anyway. We are therefore eagerly awaiting current studies (NCT02631447 / NCT03235245) on the topic of the best therapy sequence.

The size of our panel (710 genes, 2.1 MB) allows us to make a quite precise calculation of the TMB. A recent study found that a minimum size of 1.5 Mb is required to achieve accurate TMB values [23]. In addition, by sequencing both, tumor and normal tissue we can accurately detect somatic and germline variants. Shi et al. [48] compared tumor-only and matched-normal analysis pipelines and reported that tumor-only analysis falsely classified a significant number of germline variants (62%) as somatic, potentially skewing TMB towards higher values.

The extent of metastasis of the patients in our cohort was the same as in other studies, with the exception of cerebral metastases, which were otherwise excluded. Like others we had about 30% of patients with baseline liver metastases [9], about 40% of the patients had elevated baseline LDH at the beginning of combined immunotherapy and slightly more men were included [5]. Furthermore, CT evaluation was performed in the median 69 days after treatment initiation. As radiological response is usually evident within the first 12 weeks of treatment, this period seems to be appropriate [49]. The occurrence of grade 3 or 4 toxicity was 63% in our study corresponding to that of other studies [4].

Nevertheless, our results should be validated in a larger cohort and it remains unclear whether patients with low TMB and manifest liver metastases should start with a targeted therapy in the presence of a BRAF mutation instead of combined immunotherapy. Neither multivariate analysis nor control for multiple hypothesis testing were performed, so there is a risk of false discovery in assessing many variables in a non-pre-specified manner in a small dataset.

It should also be taken into account that the follow-up time of our cohort is relatively short for reliable interpretation of survival data. Further prospective and randomized studies are indispensable.

## Conclusion

Melanoma patients who are to be treated with systemic therapy should be screened and advised with regard to their individual risk factors for non-response. Presence of liver metastases and low TMB renders response to combined immunotherapy less likely. Women seem to have a disadvantage over men. Once therapy has started, > 50% increasing cell-free DNA, detectable or increasing ctDNA at first follow-up might be further indicators of non-response. The possibility of being able to detect treatment failure as early as 3 weeks after treatment initiation could become particularly important if patients suffer adverse events at an early time point of combined immunotherapy and are unsure whether to continue treatment.

Work should be done to ensure that TMB can be determined reliably and easily from a liquid biopsy, because tumor tissue is not always available and patients are not always in shape to go through surgery. Our study presents a valuable and important contribution on the way to more precision in patient selection for systemic treatment of metastasized melanoma.

## Additional file

Additional file 1: Assays, either commercially available or individually designed. (DOCX 16 kb)

Additional file 2: Impact of baseline patients’ and disease characteristics on tumor mutation burden, ctDNA and cfDNA. (DOCX 26 kb)

Additional file 3: Tumor load (computer tomography score), defined as the sum of diameters of the largest five metastases visible in CT compared to ctDNA, measured by tumor-specific variant copies/ml of plasma. Data from two assessments per patient (baseline and first follow-up CT and corresponding ctDNA). (PDF 10 kb)

## Data Availability

The datasets used and/or analysed during the current study are available from the corresponding author on reasonable request.
