# Supplementary material for: Tumor mutation burden and circulating tumor DNA in combined CTLA-4 and PD-1 antibody therapy in metastatic melanoma – results of a prospective biomarker study
Source: J Immunother Cancer. 2019 Jul 12;7:180. doi: 10.1186/s40425-019-0659-0 (PMC6625062; doi:10.1186/s40425-019-0659-0)
Supplement: Supplementary file 1 — Assays, either commercially available or individually designed. (DOCX 16 kb) [file 40425_2019_659_MOESM1_ESM.docx]

Supplemental Table 1:

Assays, either commercially available or individually designed

| **gene** | **cDNA** | **protein** | **Patients tested** | **Detectable** | **Predesigned assay** |
| --- | --- | --- | --- | --- | --- |
| **BRAF** | c.1798_1799delinsAA | p.V600K | 4 | 1 | yes |
| **BRAF** | c.1798_1799delinsAG | p.V600R | 2 | 1 | yes |
| **BRAF** | c.1799_1800delinsAA | p.V600E | 1 | 1 | yes |
| **BRAF** | c.1799T>A | p.V600E | 8 | 4 | no |
| **CDK4** | CNV | NA | 1 | 1 | no |
| **GNAQ** | c.626A>C | p.Q209P | 3 | 1 | no |
| **JAK2** | c.7A>T | p.M3L | 1 | 0 | no |
| **KRAS** | c.34G>T | p.G12C | 1 | 0 | yes |
| **KRAS** | c.351A>T | p.K117N | 1 | 1 | yes |
| **MAP2K1** | c.295_312del | p.I99_K104del | 1 | 1 | no |
| **NF1** | c.1268T>A | p.L423* | 1 | 0 | no |
| **NF1** | c.73dupA | p.T25Nfs*13 | 1 | 1 | no |
| **NRAS** | c.181C>A | p.Q61K | 2 | 2 | yes |
| **NRAS** | c.182A>G | p.Q61R | 2 | 1 | yes |
| **NRAS** | c.436G>A | p.A146T | 1 | 1 | no |
| **STAT1** | c.1945C>T | p.R649C | 1 | 0 | no |
