# Supplementary material for: Tumor mutation burden and circulating tumor DNA in combined CTLA-4 and PD-1 antibody therapy in metastatic melanoma – results of a prospective biomarker study
Source: J Immunother Cancer. 2019 Jul 12;7:180. doi: 10.1186/s40425-019-0659-0 (PMC6625062; doi:10.1186/s40425-019-0659-0)
Supplement: Supplementary file 2 — Impact of baseline patients’ and disease characteristics on tumor mutation burden, ctDNA and cfDNA. (DOCX 26 kb) [file 40425_2019_659_MOESM2_ESM.docx]

Supplemental Table 2a:

Impact of baseline patients’ and disease characteristics on tumor mutation burden

|  | **Total** | **TMB high >** **23.1** **Mut/Mb** | **TMB intermed-low ≤** **23.1** **Mut/Mb** | **P value** |
| --- | --- | --- | --- | --- |
|  | **n=30** | **n=6** | **n=24** |  |
| **Copy number [copies/ml] detection at first follow-up** | | | | |
| Not detectable | 11 | 3 | 8 | 0.662^1^ |
| Detectable | 16 | 3 | 13 |  |
| **Copy number [copies/ml] increase at first follow-up** | | | | |
| No increase | 16 | 4 | 12 | 0.617^1^ |
| Increase | 10 | 1 | 9 |  |
| **Cell-free DNA [ng/ml] at first follow-up** | | | | |
| Decrease > 50% | 6 | 3 | 3 | 0.180^2^ |
| Stable | 12 | 1 | 11 |  |
| Increase > 50% | 8 | 1 | 7 |  |
| **Targeted therapy before** | | | | |
| No | 20 | 5 | 15 | 0.633^1^ |
| Yes | 10 | 1 | 9 |  |
| **Sex** | | | | |
| Male | 15 | 6 | 9 | **0.017***^1^ |
| Female | 15 | 0 | 15 |  |
| **Liver metastasis baseline** | | | | |
| No | 20 | 6 | 14 | 0.074^1^ |
| Yes | 10 | 0 | 10 |  |
| **LDH baseline elevated** | | | | |
| No | 17 | 5 | 12 | 0.196^1^ |
| Yes | 13 | 1 | 12 |  |
| **PD-L1 Expression** | | | | |
| ≥ 1% | 8 | 1 | 7 | 0.471^1^ |
| < 1% | 9 | 0 | 9 |  |

^1^Exact Test of Fisher
^2^Exact Chi-Square Test for Trend (Monte Carlo Simulation)

*significant

Supplemental Table 2b:

Impact of baseline patients’ and disease characteristics on detectability of ctDNA measured by copy number [copies/ml] at first follow-up

|  | **Total** | **ctDNA [copies/ml]  not detectable  at first follow-up** | **ctDNA [copies/ml]  detectable  at first follow-up** | **P value** |
| --- | --- | --- | --- | --- |
|  | **n=32** | **n=14** | **n=18** |  |
| **TMB [Mut/Mb]** | | | | |
| High > 23.1 | 6 | 3 | 3 | 0.662^1^ |
| intermed-low ≤ 23.1 | 21 | 8 | 13 |  |
| **Copy number [copies/ml] at first follow-up** | | | | |
| No increase | 20 | 14 | 6 | **<0.001***^1^ |
| Increase | 11 | 0 | 11 |  |
| **Cell-free DNA [ng/ml] at first follow-up** | | | | |
| Decrease > 50% | 7 | 3 | 4 | 0.080*^2^ |
| Stable | 15 | 11 | 4 |  |
| Increase > 50% | 9 | 0 | 9 |  |
| **Targeted therapy before** | | | | |
| No | 23 | 11 | 12 | 0.694^1^ |
| Yes | 9 | 3 | 6 |  |
| **Sex** | | | | |
| Male | 19 | 8 | 11 | 1.000^1^ |
| Female | 13 | 6 | 7 |  |
| **Liver metastasis baseline** | | | | |
| No | 23 | 12 | 11 | 0.235^1^ |
| Yes | 9 | 2 | 7 |  |
| **LDH baseline elevated** | | | | |
| No | 21 | 12 | 9 | 0.061^1^ |
| Yes | 11 | 2 | 9 |  |
| **PD-L1 Expression** | | | | |
| ≥ 1% | 8 | 4 | 4 | 1.000^1^ |
| < 1% | 11 | 5 | 6 |  |

^1^Exact Test of Fisher
^2^Exact Chi-Square Test for Trend (Monte Carlo Simulation)
*significant

Supplemental Table 2c

Impact of baseline patients’ and disease characteristics on increase of ctDNA measured by copy number [copies/ml] at first follow-up

|  | **Total** | **No increase of**  **copy number**  **[copies/ml] at first**  **follow-up** | **Increase of**  **copy number [copies/ml] at first follow-up** | **P value** |
| --- | --- | --- | --- | --- |
|  | **N=31** | **n=20** | **n=11** |  |
| **Cell-free DNA [ng/ml] at first follow-up** | | | | |
| Decrease > 50% | 7 | 6 | 1 | **0.001*^2^** |
| Stable | 15 | 13 | 2 |  |
| Increase > 50% | 9 | 1 | 8 |  |
| **Targeted therapy before** | | | | |
| No | 22 | 15 | 7 | 0.683^1^ |
| Yes | 9 | 5 | 4 |  |
| **Sex** | | | | |
| Male | 18 | 11 | 7 | 0.718^1^ |
| Female | 13 | 9 | 4 |  |
| **Liver metastasis baseline** | | | | |
| No | 22 | 14 | 8 | 1.000^1^ |
| Yes | 9 | 6 | 3 |  |
| **LDH baseline elevated** | | | | |
| No | 20 | 15 | 5 | 0.132^1^ |
| Yes | 11 | 5 | 6 |  |
| **PD-L1 Expression** | | | | |
| ≥ 1% | 8 | 6 | 2 | 0.609^1^ |
| < 1% | 11 | 7 | 4 |  |

^1^Exact Test of Fisher

^2^Exact Chi-Square Test for Trend (Monte Carlo Simulation)

*significant

Supplemental Table 2d

Impact of baseline patients’ and disease characteristics on cfDNA [ng/ml] at first follow-up

|  | **Total** | **Decrease >** **50% of cell-free DNA [ng/ml] at first follow-up** | **Stable**  **cell-free DNA [ng/ml] at first follow-up** | **Increase >** **50% of cell-free DNA [ng/ml] at first follow-up** | **P value** |
| --- | --- | --- | --- | --- | --- |
|  | **n=31** | **n=7** | **n=15** | **n=9** |  |
| **Targeted therapy before** | | | | | |
| No | 22 | 5 | 12 | 5 | 0.589^2^ |
| Yes | 9 | 2 | 3 | 4 |  |
| **Sex** | | | | | |
| Male | 18 | 6 | 6 | 6 | 0.624^2^ |
| Female | 13 | 1 | 9 | 3 |  |
| **Liver metastasis baseline** | | | | | |
| No | 22 | 5 | 11 | 6 | 1.000^2^ |
| Yes | 9 | 2 | 4 | 3 |  |
| **LDH baseline elevated** | | | | | |
| No | 20 | 5 | 11 | 4 | 0.305^2^ |
| Yes | 11 | 2 | 4 | 5 |  |
| **PD-L1 Expression** | | | | | |
| ≥ 1% | 8 | 2 | 3 | 3 | 0.751^2^ |
| < 1% | 11 | 1 | 6 | 4 |  |

^2^Exact Chi-Square Test for Trend (Monte Carlo Simulation)
