# Supplementary figures and images for: Tumor mutation burden and circulating tumor DNA in combined CTLA-4 and PD-1 antibody therapy in metastatic melanoma – results of a prospective biomarker study
Source: J Immunother Cancer. 2019 Jul 12;7:180. doi: 10.1186/s40425-019-0659-0 (PMC6625062; doi:10.1186/s40425-019-0659-0)

3

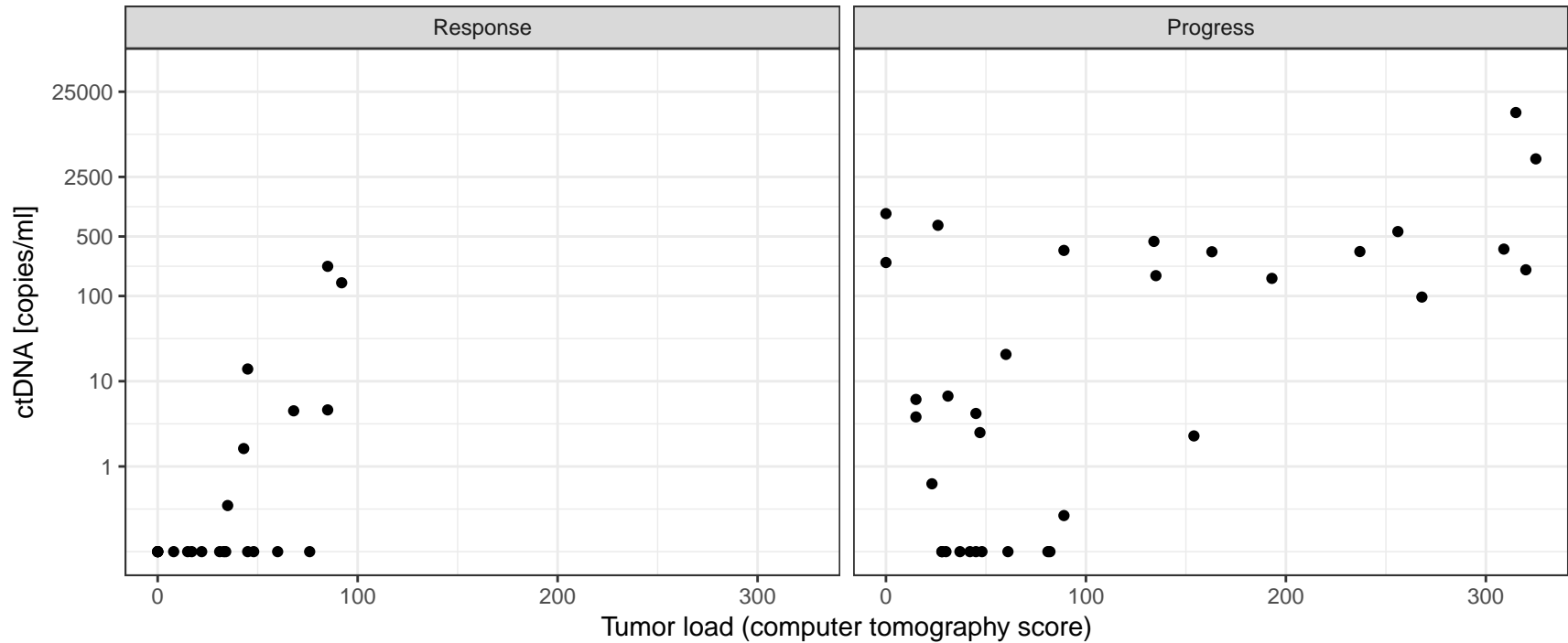

Supplement: Supplementary file 3 — Tumor load (computer tomography score), defined as the sum of diameters of the largest five metastases visible in CT compared to ctDNA, measured by tumor-specific variant copies/ml of plasma. Data from two assessments per patient (baseline and first follow-up CT and corresponding ctDNA). (PDF 10 kb) [file 40425_2019_659_MOESM3_ESM.pdf]
